# Executive Control and Associated Brain Activity in Children With Familial High-Risk of Schizophrenia or Bipolar Disorder: A Danish Register-based Study

**DOI:** 10.1093/schbul/sbad134

**Published:** 2023-09-26

**Authors:** Line Korsgaard Johnsen, Kit Melissa Larsen, Søren Asp Fuglsang, Anna Hester Ver Loren van Themaat, William Frans Christiaan Baaré, Kathrine Skak Madsen, Kristoffer Hougaard Madsen, Nicoline Hemager, Anna Krogh Andreassen, Lotte Veddum, Aja Neergaard Greve, Ayna Baladi Nejad, Birgitte Klee Burton, Maja Gregersen, Heike Eichele, Torben E Lund, Vibeke Bliksted, Anne Amalie Elgaard Thorup, Ole Mors, Kerstin Jessica Plessen, Merete Nordentoft, Hartwig Roman Siebner

**Affiliations:** Danish Research Centre for Magnetic Resonance, Centre for Functional and Diagnostic Imaging and Research, Copenhagen University Hospital, Amager and Hvidovre, Copenhagen, Denmark; Child and Adolescent Mental Health Center, Copenhagen University Hospital, Mental Health Services CPH, Copenhagen, Denmark; Danish Research Centre for Magnetic Resonance, Centre for Functional and Diagnostic Imaging and Research, Copenhagen University Hospital, Amager and Hvidovre, Copenhagen, Denmark; Child and Adolescent Mental Health Center, Copenhagen University Hospital, Mental Health Services CPH, Copenhagen, Denmark; Danish Research Centre for Magnetic Resonance, Centre for Functional and Diagnostic Imaging and Research, Copenhagen University Hospital, Amager and Hvidovre, Copenhagen, Denmark; Danish Research Centre for Magnetic Resonance, Centre for Functional and Diagnostic Imaging and Research, Copenhagen University Hospital, Amager and Hvidovre, Copenhagen, Denmark; Child and Adolescent Mental Health Center, Copenhagen University Hospital, Mental Health Services CPH, Copenhagen, Denmark; Danish Research Centre for Magnetic Resonance, Centre for Functional and Diagnostic Imaging and Research, Copenhagen University Hospital, Amager and Hvidovre, Copenhagen, Denmark; Danish Research Centre for Magnetic Resonance, Centre for Functional and Diagnostic Imaging and Research, Copenhagen University Hospital, Amager and Hvidovre, Copenhagen, Denmark; Radiography, Department of Technology, University College Copenhagen, Copenhagen, Denmark; Danish Research Centre for Magnetic Resonance, Centre for Functional and Diagnostic Imaging and Research, Copenhagen University Hospital, Amager and Hvidovre, Copenhagen, Denmark; Department of Applied Mathematics and Computer Science, Technical University of Denmark, Lyngby, Denmark; Child and Adolescent Mental Health Center, Copenhagen University Hospital, Mental Health Services CPH, Copenhagen, Denmark; Copenhagen Research Center for Mental Health, CORE, Mental Health Centre Copenhagen, Copenhagen University Hospital, Gentofte, Mental Health Services, Capital Region, Denmark; The Lundbeck Foundation Initiative for Integrative Psychiatric Research (iPSYCH), Aarhus, Denmark; The Lundbeck Foundation Initiative for Integrative Psychiatric Research (iPSYCH), Aarhus, Denmark; Department of Clinical Medicine, Faculty of Health and Medical Services, Aarhus University, Aarhus, Denmark; The Psychosis Research Unit, Aarhus University Hospital, Aarhus, Denmark; The Lundbeck Foundation Initiative for Integrative Psychiatric Research (iPSYCH), Aarhus, Denmark; Department of Clinical Medicine, Faculty of Health and Medical Services, Aarhus University, Aarhus, Denmark; The Psychosis Research Unit, Aarhus University Hospital, Aarhus, Denmark; The Lundbeck Foundation Initiative for Integrative Psychiatric Research (iPSYCH), Aarhus, Denmark; Department of Clinical Medicine, Faculty of Health and Medical Services, Aarhus University, Aarhus, Denmark; The Psychosis Research Unit, Aarhus University Hospital, Aarhus, Denmark; Medical and Science, Clinical Drug Development, Novo Nordisk A/S, Greater Copenhagen area, Denmark; Faculty of Health and Medical Sciences, Institute for Clinical Medicine, University of Copenhagen, Copenhagen, Denmark; Department of Child and Adolescent Psychiatry, Copenhagen University Hospital, Psychiatry Region Zealand, Roskilde, Denmark; Child and Adolescent Mental Health Center, Copenhagen University Hospital, Mental Health Services CPH, Copenhagen, Denmark; Copenhagen Research Center for Mental Health, CORE, Mental Health Centre Copenhagen, Copenhagen University Hospital, Gentofte, Mental Health Services, Capital Region, Denmark; The Lundbeck Foundation Initiative for Integrative Psychiatric Research (iPSYCH), Aarhus, Denmark; Division of Psychiatry, Regional Resource Centre for Autism, ADHD and Tourette syndrome Western Norway, Haukeland University Hospital, Bergen, Norway; Center of Functionally Integrative Neuroscience, Aarhus University Hospital, Aarhus, Denmark; Center of Functionally Integrative Neuroscience, Aarhus University Hospital, Aarhus, Denmark; The Lundbeck Foundation Initiative for Integrative Psychiatric Research (iPSYCH), Aarhus, Denmark; Department of Clinical Medicine, Faculty of Health and Medical Services, Aarhus University, Aarhus, Denmark; The Psychosis Research Unit, Aarhus University Hospital, Aarhus, Denmark; Faculty of Health and Medical Sciences, Institute for Clinical Medicine, University of Copenhagen, Copenhagen, Denmark; Child and Adolescent Mental Health Center, Copenhagen University Hospital, Mental Health Services CPH, Copenhagen, Denmark; The Lundbeck Foundation Initiative for Integrative Psychiatric Research (iPSYCH), Aarhus, Denmark; Department of Clinical Medicine, Faculty of Health and Medical Services, Aarhus University, Aarhus, Denmark; The Psychosis Research Unit, Aarhus University Hospital, Aarhus, Denmark; Division of Child and Adolescent Psychiatry, Department of Psychiatry, The University Hospital of Lausanne (CHUV) and University of Lausanne, Lausanne, Switzerland; Child and Adolescent Mental Health Center, Copenhagen University Hospital, Mental Health Services CPH, Copenhagen, Denmark; The Lundbeck Foundation Initiative for Integrative Psychiatric Research (iPSYCH), Aarhus, Denmark; Faculty of Health and Medical Sciences, Institute for Clinical Medicine, University of Copenhagen, Copenhagen, Denmark; Copenhagen Research Center for Mental Health, CORE, Mental Health Centre Copenhagen, Copenhagen University Hospital, Gentofte, Mental Health Services, Capital Region, Denmark; The Lundbeck Foundation Initiative for Integrative Psychiatric Research (iPSYCH), Aarhus, Denmark; Danish Research Centre for Magnetic Resonance, Centre for Functional and Diagnostic Imaging and Research, Copenhagen University Hospital, Amager and Hvidovre, Copenhagen, Denmark; Faculty of Health and Medical Sciences, Institute for Clinical Medicine, University of Copenhagen, Copenhagen, Denmark; Department of Neurology, Copenhagen University Hospital Bispebjerg, Copenhagen, Denmark

**Keywords:** functional magnetic resonance imaging, task-related BOLD response, visuomotor response conflict, flanker task, neurocognitive functioning, genetic predisposition, endophenotype, cohort study

## Abstract

**Background and Hypotheses:**

Impaired executive control is a potential prognostic and endophenotypic marker of schizophrenia (SZ) and bipolar disorder (BP). Assessing children with familial high-risk (FHR) of SZ or BP enables characterization of early risk markers and we hypothesize that they express impaired executive control as well as aberrant brain activation compared to population-based control (PBC) children.

**Study Design:**

Using a flanker task, we examined executive control together with functional magnetic resonance imaging (fMRI) in 11- to 12-year-old children with FHR of SZ (FHR-SZ) or FHR of BP (FHR-BP) and PBC children as part of a register-based, prospective cohort-study; The Danish High Risk and Resilience study—VIA 11.

**Study Results:**

We included 85 (44% female) FHR-SZ, 63 (52% female) FHR-BP and 98 (50% female) PBC in the analyses. Executive control effects, caused by the spatial visuomotor conflict, showed no differences between groups. Bayesian ANOVA of reaction time (RT) variability, quantified by the coefficient of variation (CV_RT_), revealed a group effect with similarly higher CV_RT_ in FHR-BP and FHR-SZ compared to PBC (BF_10_ = 6.82). The fMRI analyses revealed no evidence for between-group differences in task-related brain activation. Post hoc analyses excluding children with psychiatric illness yielded same results.

**Conclusion:**

FHR-SZ and FHR-BP at age 11–12 show intact ability to resolve a spatial visuomotor conflict and neural efficacy. The increased variability in RT may reflect difficulties in maintaining sustained attention. Since variability in RT was independent of existing psychiatric illness, it may reflect a potential endophenotypic marker of risk.

## Introduction

Schizophrenia (SZ) and bipolar disorder (BP) are severe and heritable^1^ psychiatric disorders.^2^ Children of parents with SZ or BP (ie, familial high-risk; FHR) have twice as high-risk for developing a severe mental disorder compared to the general population^3^ together with a wide range of other mental disorders before adulthood.^4^ Therefore, investigations in children with FHR of SZ or BP can contribute to the identification of factors of early neurodevelopmental vulnerability. Executive control represents the capability to withhold inappropriate responses and to detect and filter out irrelevant or conflicting information to the task at hand.^5,6^ Effective executive control relies on the integrity of the multiple demand network that mediate information, attention, and inhibitory processing, including areas of the anterior cingulate cortex (ACC), sub-areas of the parietal cortex, the premotor area, and the insula.^7^ Impaired executive control leads to impulsive behaviors which are common in individuals with neurodevelopmental disorders, including SZ, BP, and attention-deficit/hyperactivity disorder (ADHD). Impaired visuospatial-motor mapping in the context of interfering spatial instruction cues and associated changes in task-related brain activity have been reported in functional magnetic resonance imaging (fMRI) studies in individuals diagnosed with SZ^8,9^ and BP,^8,10^ as well as in individuals at clinical high-risk of SZ^9^ or BP,^11^ and adults with FHR,^12–19^ demonstrating its’ potential as a prognostic and endophenotypic marker of severe mental disorder. This potential of executive control is also highlighted by the Research Domain Criteria Initiative (RDoc; see eg, the Response Selection sub-construct of the Research Domain Criteria Matrix; https://www.nimh.nih.gov/research/research-funded-by-nimh/rdoc/constructs/cognitive-control, accessed May 26, 2023). However, only a limited number of neurobiological investigations of young individuals (age <18) exist, leaving out information on early developmental aspects of the FHR state. The studies show that young individuals (age 8–25 years) with FHR of severe mental disorder display aberrant task-related brain activation in fMRI studies investigating selective attention and/or executive control.^20–23^ Yet, sample sizes are small, and results between studies are divergent which impedes finite conclusions.^24^ This has motivated The Danish High Risk and Resilience Study to investigate the largest-to-date, representative, prospective cohort of age matched children with and without FHR of SZ or BP.^25,26^

In the first wave of the Danish High Risk and Resilience Study, The VIA 7-study, 7-year-old children with FHR for SZ and BP exhibited behavioral neurocognitive impairments^27–30^ that persisted as a stable developmental deficit at age 11.^31^ Specifically, executive control was impaired along with higher variability in reaction times (RTs) at age 7 in children with FHR of SZ compared to controls.^27^ The impairment in executive control at age seven may constitute an early endophenotype for the later development of SZ and BP.^32^ However, little is known about the functional properties of the neural networks mediating executive control impairments in children with FHR of SZ or BP during the pre-pubertal neurodevelopmental period.

In the first follow-up of the VIA 7-study, The VIA 11 study,^26^ we have assessed the cohort, now age 11, using the Flanker task in neuroimaging settings of electro-encephalography^33^ and fMRI. For the present paper we use the blood-oxygen level dependent (BOLD) signal obtained with fMRI to probe functional brain activation during executive control in the VIA 11 cohort. We hypothesized based on the findings from the VIA 7-study,^27,34^ that children with FHR-SZ and FHR-BP would have difficulties in solving the visuomotor response conflict induced by incompatible visuospatial cues. Further, we expected that FHR-SZ and FHR-BP children would display aberrant neural engagement of brain regions within the multiple demand network compared with PBC, based on previous studies.^20–23^ The flanker-EEG analyses will be presented in a separate paper.

## Methods

For a detailed description of data acquisition and analyses procedures see the eMethods.

### Participants

Children were recruited through The Danish High-Risk and Resilience study—VIA 11, including children with at least one parent with SZ (ie, FHR-SZ) or BP (ie, FHR-BP), and children with parents without these two disorders (ie, PBC). The cohort and overall study design are detailed elsewhere.^25,26^

Written informed consent was obtained from the parent or legal guardian of the child. Children received gift cards for their participation. The VIA 11 study was approved by the National Committee on Health Research Ethics (Protocol number: H 16043682) and the Danish Data Protection Agency (ID number RHP-2017-003, I-suite no. 05333) and conducted in accordance with the Declaration of Helsinki.

### Data Acquisition

The children completed one ~10-min session of a modified arrow-version of the Eriksen flanker task^35,36^ with an equal amount of congruent and incongruent trial conditions (eFigure 2 in eMethods). The task was completed during fMRI at 3 T to examine executive control-related brain activation. Preprocessing of fMRI data is described in eMethods section “*MRI acquisition and preprocessing*”.

Clinical assessment of the children was obtained on a separate day from the MRI session. See “*Clinical Measures*” section in the supplementum.

### Behavioral Outcome Measures

The behavioral consequences of the visuomotor response conflict caused by spatially incompatible flanker stimuli were characterized by response accuracy (resp-acc) defined as the percentage of correct responses, mean reaction time (RT) defined as the time from the appearance of the target arrow to the button press in trials with correct responses, and global RT variability (RT_CV_) defined as the standard deviation of RT divided by mean RT. Mean values were calculated for congruent and incongruent trials separately. To assess the flanker effect, we computed the difference between incongruent and congruent trials for resp-acc (denoted Δ_resp-acc_), RT (denoted Δ_RT_), and RT_CV_ (denoted Δ_RTCV_). For a detailed description of the behavioral outcome measures, please see the “*Behavioral outcome measures, data analysis, and statistical inference*” section in the eMethods. Additionally, we tested for between-group differences in the speed-accuracy trade-off. To accomplish this, we employed an RT distribution-analytical technique in which all trials were sub-divided into four clusters at the within-subject level, assigning them to one of four RT quartiles.^37^ Mean values were computed for each RT quartile and denoted as time-bin 1–4. Time-bin 1 included all trials belonging to the 25% fastest responses. Conversely, time-bin 4 comprised the 25% slowest trials of each subject. Time-bin 1 thus represents behavior during fast, impulsive responses, whereas time-bin 4 represents behavior during slow, deliberate responses. Time-bins 2 and 3 lie in-between these two extremes. Since the flanker task introduced a visuospatial conflict between the spatial cue and the required motor response, time-bins were separately defined for overall faster congruent and overall slower incongruent trials. Several behavioral measures (eg, resp-acc, RT, Δ_resp-acc_, and Δ_RT_) were subjected to RT distribution-analysis. This enabled us to test for between-group differences in task performance associated with the temporal RT dynamics and the presence or absence of a visuospatial response conflict.^37^

### Statistical Analyses

Statistical tests on behavioral and clinical outcome measures were performed in SPSS (IBM SPSS Statistics version 25, release 25.0.0.2) and JASP (JASP Team [2020], Version 0.8.1).^38^ We used Bayesian ANOVA for testing group differences on the clinical measures. In case of non-normal distributed data, we used the non-parametric Kruskal–Wallis or Pearson chi-square test with three groups, see table 1 and eTable 5 in eMethods for details. Bayesian repeated measures ANOVAs were used for testing group differences on the behavioral outcome measures of RT and CV_RT_. Models included Group as between-subject factor with three levels (FHR-SZ, FHR-BP, and PBC) and Condition as within-subject factor with two levels (congruent, incongruent). Δ_resp-acc_, Δ_RT_ and Δ_CVRT_ were tested for group differences by separate Bayesian ANOVAs with Group as fixed effect.

**Table 1.** Demographic and clinical characteristics of the included children

	Total	FHR-BP	FHR-SZ	PBC	Bayes Factor_10_^d^			
					Analysis of Effects	Post Hoc Comparisons		
					Group	FHR-BP vs FHR-SZ	FHR-BP vs PBC	FHR-SZ vs PBC
Children, *n*	246	63	85	98	—	—	—	—
Females, *n* (%)	119 (48.4)	33 (52)	37 (44)	49 (50)	0.52^f^	—	—	—
DRCMR test-site, *n* (%)	117 (47.6)	32 (50)	45 (53)	40 (41)	0.22^g^	—	—	—
CFIN test-site, *n* (%)	129 (52.4)	31 (49)	40 (47)	58 (59)		—	—	—
Age at scan, mean years (SD)	12.1 (0.3)	12.1 (0.3)	12.1 (0.3)	12.1 (0.3)	0.1	—	—	—
Handedness, *n* (%) right-handed^a^	209 (85)	53 (84)	69 (81)	87 (89)	—	—	—	—
CBCL^b^					—			
CBCL, Total score, mean (SD)	17 (19)	20 (21)	22 (21)	12 (13)	BF_10_ > 100	0.2	9.1	>100
CBCL, Externalizing score, mean (SD)	4 (5)	5 (6)	5 (7)	2 (3)	BF_10_ > 100	0.2	26.8	>100
CBCL, Internalizing score, mean (SD)	6 (6)	7 (7)	6 (6)	5 (5)	1.6	—	—	—
CGAS, mean score (SD)^c^	72 (15)	70 (15)	68 (15)	76 (14)	19.1	0.3	1.7	30.7
Any Axis-I disorder, *n* (%)^e^	103 (42)	32 (51)	44 (52)	27 (28)	0.001^f^	—	—	—
ADHD, *n* (%)^e^	32 (13.0)	6 (9.5)	16 (18.8)	10 (10.2)	0.123^g^	—	—	—

Demographic and clinical characteristics of 85 children at familial high-risk of schizophrenia (FHR-SZ), 63 children at familial high-risk of bipolar disorder (FHR-BP), and 98 population-based control (PBC) children from the Danish High Risk and Resilience Study—VIA 11.

DRCMR, Danish Research Center for Magnetic Resonance; CFIN, Center of Functionally Integrative Neuroscience; SD, standard deviation; CBCL, Child-behavior checklist; CGAS, Child Global Assessment Scale.

^a^Handedness was assessed with the Edinburgh handedness inventory^33^ (see “*Clinical Measures*” section in eMethods) and data is presented as % right-handed according to the laterality quotient score.

^b^Two participants (1.4%) did not complete the CBCL. Higher scores indicate more problem behavior on the total score as well as on the two broad-band subscales; Internalizing and Externalizing scores.

^c^Higher scores (0–100) on the CGAS indicate higher global level of functioning.

^d^Bayes Factors (BF) where estimated with Bayesian analyses of variance (ANOVA) in favor of an effect of group (the alternative hypothesis), that is, BF_10_, using default priors and 1000 iterations (see “Methods” section).

^e^Quantification of any Axis-I disorder was obtained with the Schedule for Schedule for Affective Disorders and Schizophrenia for School-Age Children-Present and Lifetime Version (K-SADS-PL) and depicts number of children with any past or present Axis-I diagnosis excluding elimination disorders. 244 children completed the questionnaire.

^f^Pearson chi-square test of independence.

^g^Kruskal–Wallis test.

^h^Quantification of Attention-Deficit/hyperactivity Disorder (ADHD) was obtained with the K-SADS-PL and depicts number of children with any past or present ADHD diagnosis.

Resp-acc data for congruent and incongruent trials, as well as in the RT distributional analyses, violated tests for normality and inference on resp-acc was therefore explored using automated non-parametric testing (Kruskall–Wallis) for independent samples in SPSS.

Normal distributed data for the RT distributional analysis (Dist-Δ_resp-acc_ and Dist-Δ_RT_) were analyzed with separate Bayesian repeated measures ANOVAs including Dist-Δ_resp-acc_ and Dist-Δ_RT_, respectively, as repeated measures factor with four levels (RT bin 1–4). Group was entered as a between-subject factor with three levels (FHR-BP, FHR-SZ, HC). Age, sex, and test-site were entered as covariates in all analyses. If evidence for effects of single covariates were present in the Bayesian analyses, analyses were rerun, including covariates as between-subject factors for inference on group by covariate effects. Group effects that met evidence for the alternative hypothesis larger than moderate were tested with post hoc comparisons with null control. Common frequentist statistics make binary inferences in terms of “significance” based on a *P*-value and a pre-specified statistical threshold. Conversely, Bayesian statistical estimates the relative level of evidence in favor or against the alternative hypothesis, expressed by the Bayes factor (BF). Further details on the interpretation of the BF can be found in the eMethods section “*Behavioral outcome measures, data analysis and statistical inference*”.

fMRI data were analyzed using a general linear model (GLM) in SPM12 (Wellcome Centre for Human Neuroimaging) run in MATLAB (The MathWorks, Inc., version R2020a update 3). See eMethods section “*Statistical analyses*” for a full account of the design. At the subject level a main incongruent > congruent contrast was formed based on task regressors and used to model successful executive control activation, as we only included trials in which the participants answered correctly. At the group-level, the main contrasts were analyzed in a univariate flexible factorial ANOVA with group (FHR-BP, FHR-SZ, PBC) as between-subject factor. In accordance with Woo et al.^39^, we applied a cluster-forming threshold of *P* < .001, uncorrected, and a Gaussian random field-based family wise error (FWE) correction of *P* < .05 at the cluster level for reporting of significant clusters of brain activation. Regions of interest (ROIs) were defined as spheres with 10 mm radius centered at the peak activation coordinates from the whole-brain analysis testing the main effect of successful executive control (ie, incongruent > congruent contrast) across groups.

To test for a linear relationship between brain activity and increasing RT time, we sorted all responses in quartiles depending on the RT with bin1 including the 25% fastest responses and bin 4 comprising the 25% slowest responses. The RT bin quartiles were included as a first parametric modulator of interest in the first-level GLM. Brain-behavior correlations were performed on behavioral variables showing group effects. The fMRI analyses included age, sex, handedness, and test-site as covariates.

Additionally, we analyzed mean parameter estimates from the main contrast in ROI analyses within the Bayesian framework to characterize evidence for or against the alternative hypothesis. ROIs were defined as 10 mm spheres with their center placed according to peak activation coordinates (cluster-level FEW-corrected *P* < .05) from the main effect of the successful executive control activation analysis across groups. ROIs were included as a repeated measures factor with nine levels (ROI I, ROI II, etc.) and group as between-subject factors with three levels (ie, FHR-BP, FHR-SZ, PBC) in a Bayesian repeated measures ANOVA implemented in JASP.^38^

## Results

Demographic and clinical characteristics as well as the statistical test results of included children are presented in table 1. The final sample consisted of 246 children: 85 children (44% female) with FHR-SZ, 63 children (52% female) with FHR-BP and 98 (50% female) PBC. The three groups were comparable on age (BF_10_ = 0.1, moderate evidence against a group effect), handedness (BF_10_ = 0.09, strong evidence against group effect), and sex (Pearson chi-square, *P* = .52). Children with FHR-SZ and FHR-BP presented with higher behavioral problem scores on the total score and on the externalizing score compared to PBC (total score; FHR-SZ vs PBC: BF_10_ > 100, FHR-BP vs PBC: BF_10_ = 9.1. Externalizing score; FHR-SZ vs PBC: BF_10_ > 100, FHR-BP vs PBC: BF_10_ = 26.8), and lower global functioning scores for FHR-SZ compared to PBC (BF_10_ = 30.7). The behavioral problem score nor the global functioning score differed between FHR-SZ and FHR-BP. There was higher incidence of Axis-I disorders in both the FHR groups compared to PBC (Pearson chi-square, *P* < .001), however the number of ADHD diagnoses was similar between groups (Kruskal–Wallis, *P* = 0.123).

### Behavioral Executive Control

**Fig. 1.**
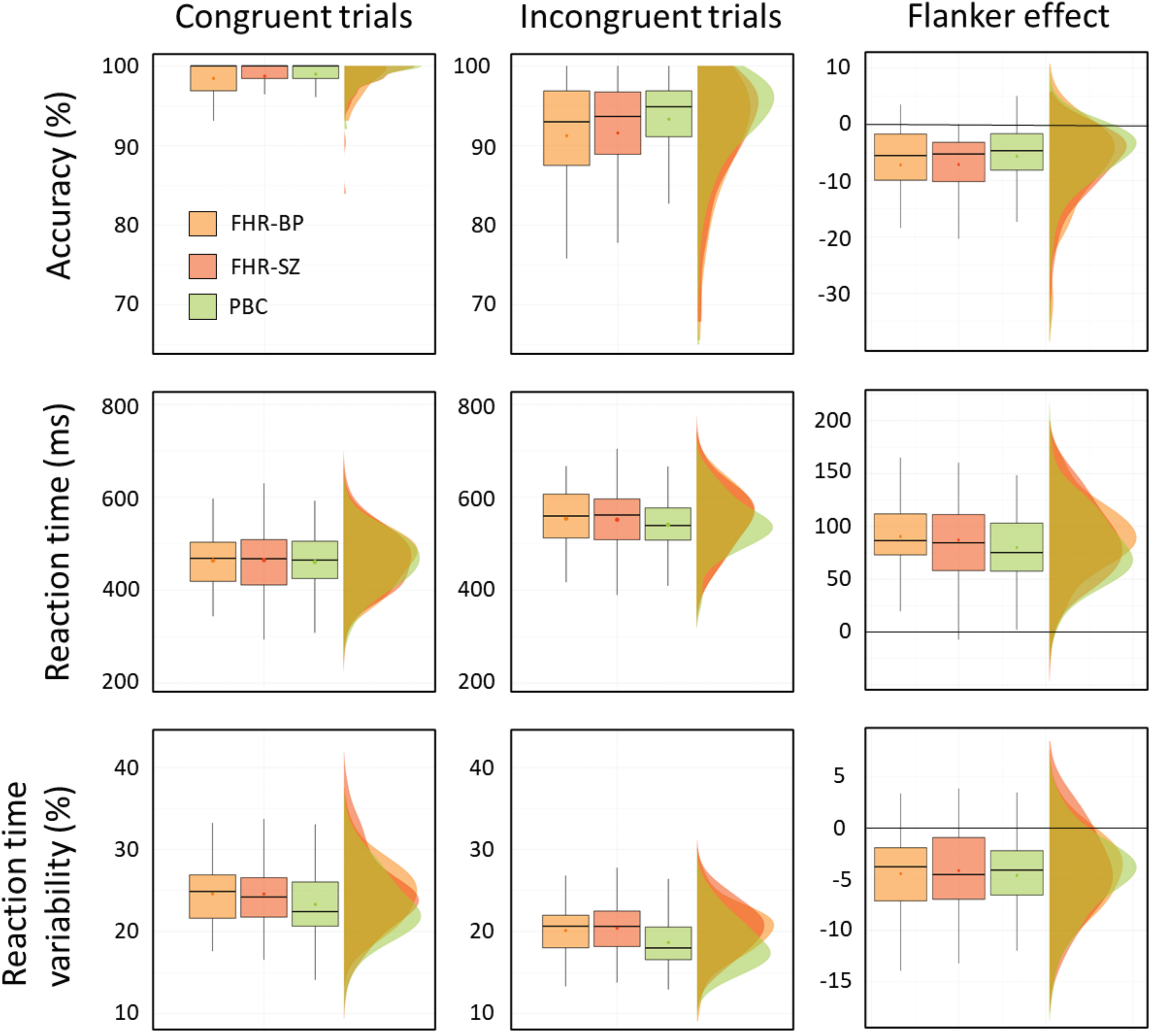
Task performance measures, that is, response accuracy, reaction time and reaction time variability on the modified arrow-version of the Eriksen flanker task for children at familial high-risk (FHR) of schizophrenia (FHR-SZ; dark orange), bipolar disorder (FHR-BP; light orange), or neither of these disorders (population-based controls [PBC]; green). Data has been plotted as typical boxplots illustrating the minimum, maximum, median, first quartile, and third quartile, as well as the data distribution clouds to match. The dot within each boxplot illustrates the mean value. Performance across the three groups shows an overall decrease in accuracy rate (top horizontal panel), and an overall increase in reaction time (middle horizontal panel), from congruent (left vertical panel) to incongruent (middle vertical panel) trial conditions as well as the difference (ie, the flanker effect) in these measures (right vertical panel). Values larger than zero indicate an increase in performance measure from incongruent to congruent conditions whereas values lower than zero indicate a lower performance measure. The bottom horizontal panel shows reaction time variability, calculated as the coefficient of variation (CV=σ{RT}/μ{RT}⋅100%) in which there was moderate evidence (Bayes factor [BF]_10_ = 6.8) for an effect of group but moderate evidence against (BF_10_ = 0.22) a group by condition (ie, congruent and incongruent) interaction. ms; milliseconds, FHR-SZ; Familial high-risk of schizophrenia, FHR-BP; Familial high-risk of bipolar disorder, PBC; Population-based controls.

The three groups performed similarly on resp-acc across conditions (*P* = .218, congruent, *P* = .135, incongruent), with all groups showing higher accuracies on congruent trials compared to incongruent trials (figure 1, top row, and supplemental eTable 5). All groups showed slower RT for incongruent trials compared to congruent trials (BF_10_ > 100) with similar mean values (BF_10_ = .22), see figure 1, middle row. Across the two conditions the variability of response timing, RT_CV_, differed between groups (BF_10_ = 6.88) indicating that children with FHR-SZ and children with FHR-BP showed higher RT_CV_ compared to PBC, figure 1 bottom row. Sex and test-site showed strong (BF_10_ = 19.9) and decisive (BF_10_ > 100) effects on RT_CV_, respectively. Adding these as between-subject factors revealed no interaction with the group effect.

**Fig. 2.**
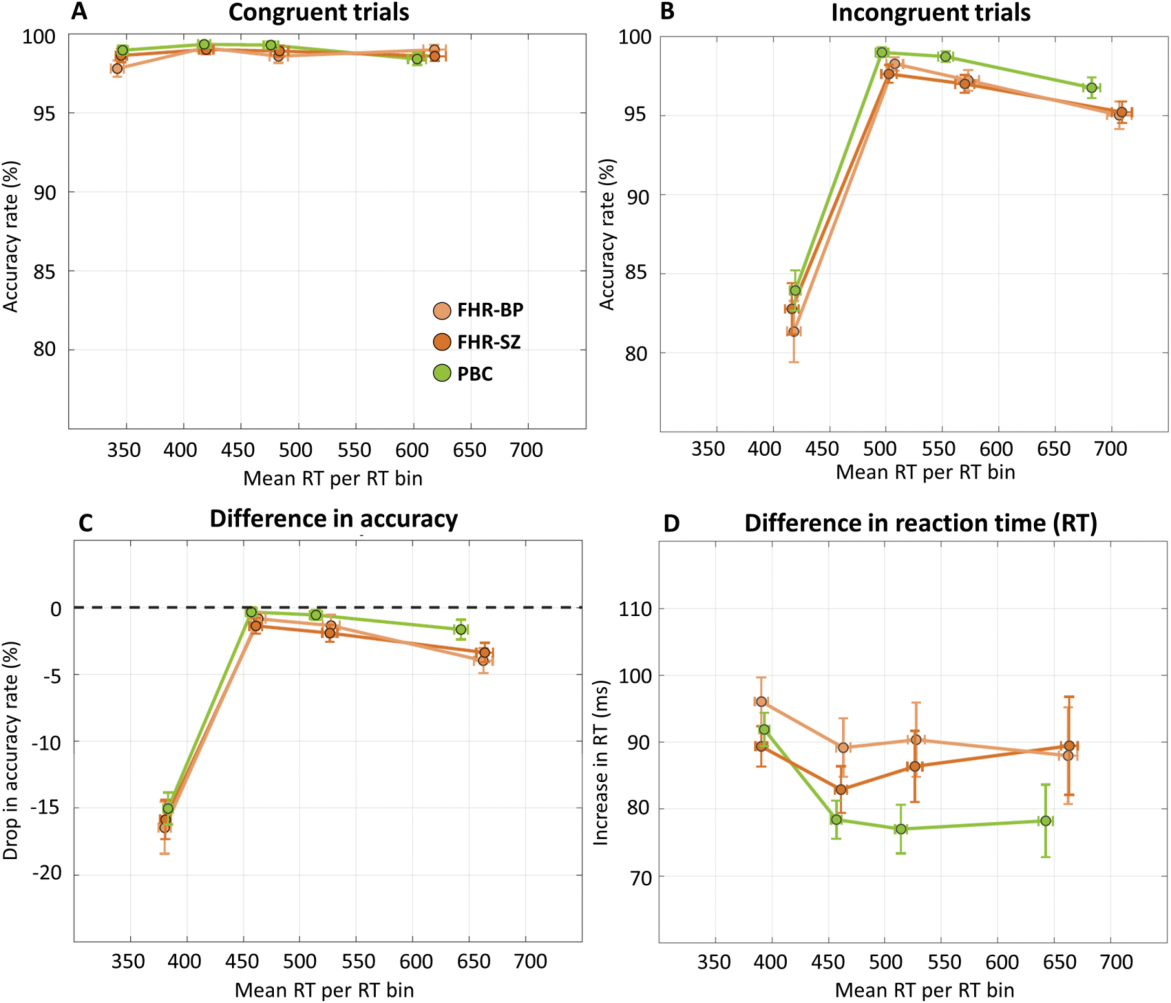
Reaction time (RT) distribution-analysis of response accuracy rate on congruent (A) and incongruent (B) trials (Dist-resp-acc). Delta plots show the magnitude of flanker effects on response accuracy rate (C; Dist-Δ_resp-acc_) and RT (D; Dist-Δ_RT_) as a function of RT on the modified arrow-version of the Eriksen flanker task for children at familial high-risk of schizophrenia (FHR-SZ), bipolar disorder (FHR-SZ) or neither of these disorders (population-based controls; PBC). Mean RTs are plotted according to the 1st, 2nd, 3rd, and 4th quartile of RTs according individual RT distributions for correct-proceeded-by-correct trials (see eMethods section “*Behavioral outcome measures, data analysis and statistical inference*”). Error bars indicate standard error of the mean (SEM) calculated by $\sigma /\sqrt{n}$ and illustrated for both mean RTs of the quartiles as well as the accuracy rates (A–C) and the drop in RT (D).

The speed-accuracy trade-off analysis revealed an effect of time-bin for the congruent (Friedman’s ANOVA, *P* = .034) and incongruent (Friedman’s ANOVA, *P* < .001) condition, showing higher accuracies in the slowest, most deliberate trials. This trade-off was similar across all three groups, figure 2B. Groups did not differ on the Flanker effect for either accuracy (Dist-Δ_resp-acc_ BF_10_ = .11) or RT (Dist-Δ_RT_ BF_10_ = .15), figure 2C and D, respectively.

### Executive Control-related Brain Activation

**Fig. 3.**
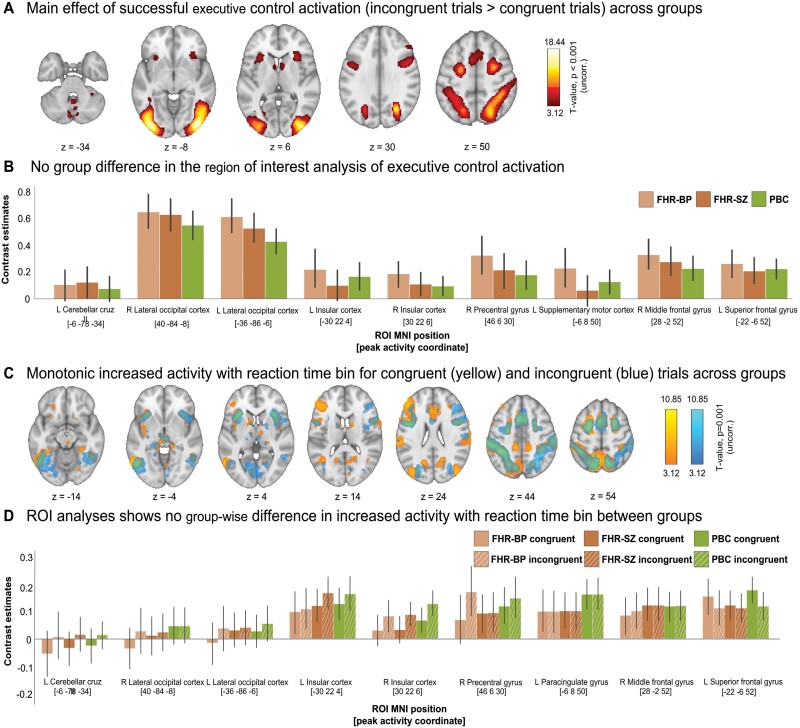
Executive control-related brain activation results from 63 children with familial high-risk (FHR) of bipolar disorder (FHR-BP), 85 children with FHR of schizophrenia (FHR-SZ) and 98 population-based control children without FHR of these disorders. (A) The group-level whole-brain analysis on the contrast between correct incongruent trials larger than correct congruent trials (successful executive control activation) showed significant increased activation in nine task-related clusters (cluster-level FWE-corrected *P* < .05) within areas of cerebellum, left and right lateral occipital cortex, left and right insular cortex, right precentral cortex, left supplemental motor area, middle frontal gyrus, and superior frontal gyrus. The color bar indicates *T*-values. The analysis did not show any significant group differences or any significant condition by group interactions. (B) Bar plot showing contrast estimates (CE) from the region of interest (ROI) analysis on the successful executive control activation. Error bars indicate the standard deviation. (C) Whole-brain activation modulated by reaction time (RT) bin on correct congruent trials larger than 0 (yellow) and correct incongruent trials larger than zero (blue) across groups. There was no significant between-group difference. (D) Bar plot showing CE from the ROI analysis on the brain activation modulated by RT bin analysis. Congruent CE are solid bars, incongruent contrast estimated are scratched bars. Error bars indicate the standard deviation. Incongruent CE are shown with hatched bars.

To ensure that our task engaged the relevant executive control network, we investigated the main congruency effect on brain activity across groups. In accordance with the task-related activation patterns that have been reported in two previous meta-analyses,^7,40^ we found consistent task-related activations in brain regions supporting executive control. The activation pattern comprised nine clusters across the three groups (figure 3A, and eTable 2 in eMethods): the left cerebellar crus II, the inferior division of right and left lateral occipital cortex, left and right insula, right precentral gyrus, left supplementary motor area (SMA), right middle frontal gyrus (MFG), and left superior frontal gyrus (all *P* < .05, cluster-level corrected). We found no significant main effect of group in the whole-brain analyses regarding the main congruency effect. This was confirmed in the ROI analysis using Bayesian inference (figure 3B, and eTable 6 A in eMethods).

The speed-accuracy trade-off analysis including time-bin as a first parametric modulation of interest revealed 10 significant clusters of increased activation with increased time-bin (ie, slower RTs) spanning insular, frontotemporal, striatal, thalamic, and cingulate brain areas for the congruent condition (figure 3C, and eTable 3 in eMethods). For the incongruent condition nine significant clusters of increased activation with increased time-bin (ie, slower RTs) were located within temporoparietal, thalamic, striatal, and occipital brain areas (figure 3C, and eTable 3 in eMethods). This network was stable across groups and confirmed in a follow-up ROI analysis using Bayesian inference (figure 3D, and eTable 6B and C in eMethods).

**Fig. 4.**
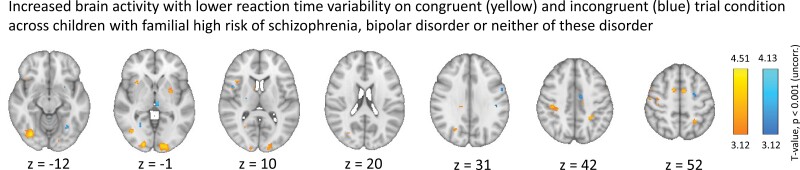
Whole brain blood-oxygen level-dependent (BOLD) response with reaction time (RT) variability—calculated as the coefficient of variation (CV)—as linear regressor of interest across 63 children at familial high-risk (FHR) of bipolar disorder (FHR-BP), 85 children with FHR of schizophrenia (FHR-SZ), and 98 children without FHR of these disorders (population-based controls). Contrast images are based on correct responses on congruent trial events > 0 (yellow) and incongruent trials events > 0 (blue) and show brain activation as a function of lower reaction variability—a reaction time stability network that is.

To follow-up on the behavioral findings of moderate evidence for an effect of group on RT variability, RT_CV_ was added as a regressor of interest in two independent second level analyses. We entered RT_CV_ for congruent and incongruent trials and used the contrast of congruent > 0 and incongruent > 0 from the first level, respectively. For the congruent contrast, we found a significant negative relationship between RT_CV_ and brain activity in four clusters within occipital and parietal brain areas across the three groups (figure 4, supplementary eTable 4). This network was similar across groups.

To disentangle effects of FHR from that of existing psychiatric diagnoses, we replicated all analyses excluding all children with any past or present Axis-I diagnosis (*n* = 103). These post hoc analyses resulted in similar findings as the ones reported in the total sample.

## Discussion

We show evidence that children with FHR for SZ or BP express higher variability in RT during a spatial visuo-motor response conflict task, probing executive control. This variability persisted when excluding children with any past or present psychiatric diagnosis. We therefore argue that this increased variability in response timing might reflect an endophenotypic trait for risk of SZ and BP. The behavioral fingerprint was not accompanied by altered group-wise brain responses, indicating a similar brain network engagement when solving the flanker task.

The increased RT variability, indexed by a higher RT_CV_, was observed in children with FHR-SZ and FHR-BP independently of the presence or absence of a visuospatial response conflict. A more variable timing of responses in the FHR groups agrees with previous results from this cohort, observed at age 7.^27,28^ It also extends earlier findings in children with FHR of SZ or BP beyond those already reported in this cohort across different paradigms, testing attentional, and executive control abilities.^17,23,41^ Indeed, larger variability of RT is shared across many disorders such as ADHD, SZ and BP^42–44^ and refers to an increased within-subject fluctuation of trial-to-trial behavior making it an attentional attribute related to the maintenance of continuous performance.^45^

In addition to the increased variability in RT at age 7, the children with FHR in the VIA 7-study showed impaired executive control (ie, response accuracy [resp-acc] and mean RT)^27^ which was hypothesized to still be present at age 11. Indeed, separate analyses of 23 neurocognitive measures in this cohort, spanning multiple domains from age 7 to 11 showed a stable developmental deficit for the children with FHR of SZ compared to controls.^31^ However, we found evidence against between-group differences in executive control at age 11–12. This finding suggests that impaired executive control may be of transitory character, or it may be attributed to methodological factors. The flanker paradigm used at age 7 was run at a faster pace with relatively short inter-trial interval (ITI; 900 ms) and applied in a larger sample size (*n* = 492). In our fMRI study, we implemented a slower version of the task to accommodate the sluggishness of the hemodynamic response. The longer ITIs may have resulted in a less demanding paradigm. It is thus possible that the high-risk groups would have maintained impaired performance in a more challenging setting with shorter ITIs.

Meta-analytical evidence, based on samples with broad age ranges that may obscure neurocognitive developmental factors, have shown impaired executive control using Stroop tasks in young (age 15–29) individuals with FHR of SZ^46^ with small to medium effect sizes (Cohen’s *d* = 0.28 [95% CI 0.04–0.52]), and in youth (mean age 10–25) with FHR of BP^47^ with similar effect sizes (Cohen’s *d* = 0.3 [95% CI 0.11–0.48]) when compared to controls. The Stroop task is, like the Flanker task, used to assess executive functions.^48,49^ However, intact executive control measured with Stroop in child and adolescent offspring of patients diagnosed with schizophrenia or bipolar disorder at baseline (age: 10–12 years) and follow-up (age: 12–14 years) have also been shown,^50^ leaving this cognitive measure yet unresolved as a candidate endophenotype in young FHR populations.

Our task reliably evoked executive control-related brain activation in areas typically associated with executive control, that is, cerebellar, occipital, parietal, premotor, and prefrontal areas.^7,40,51–54^ Within this network, task-related brain activation was comparable between our three groups. In child FHR populations, fMRI investigations of task-related activation together with executive control are scarce.^15,24^ To the best of our knowledge, this is the first task-based fMRI study on executive control in children with FHR of SZ. Tapping into executive control, a small fMRI study examined task-related activation during a stop-signal task in 13 adolescents with FHR of BP (mean age ~13 years).^20^ The study reported significantly greater activation in putamen during unsuccessful motor inhibition compared to children without FHR (*n* = 24, mean age ~14).^20^ Another study on cognitive flexibility (using a change task), reported that youth with FHR of BP (*n* = 13, mean age ~14 years) showed increased activity in ventrolateral prefrontal and inferior parietal areas during successful cognitive flexibility and increased brain activation in the caudate nucleus during failed change trials compared to controls (*n* = 21, mean age ~14).^22^ Together these studies point to hyper-activity of dorso-striatal structures as a mechanism for failing inhibitory processes in children with FHR of BP. However, the sample sizes of these two studies are very small, bearing a considerable risk of false positive findings. In addition, the focus on unsuccessful inhibition makes comparison to the current investigation challenging. In our setup, most of the children completed the task without mistakes. Their performance thus limits us from assessing unsuccessful executive control.

Our study has strengths and weaknesses. The present study includes the largest number of children (>100) with FHR of SZ or BP at a similar young age of 11–12. Throughout adolescence, executive control continues to improve until full maturation in adulthood.^55–57^ Therefore, the mean age of the children in our cohort represents a developmentally sensitive time during which continuous brain changes in relation to maturation and behavioral performance occur in parallel.^58^ Using a sample with a narrow age range reduces the risk of obscuring age effects of neurocognitive and brain maturation in the event of a developmental lag.^59^ However, the pre-adolescent age-group may also leave our analysis too early in development to detect neurobiological differences that may arise later in life.

Tasks that probe aspects of executive control, such as the flanker task, may be influenced by other cognitive processes. While the flanker task is designed to probe executive control, other aspects of cognition are also engaged such as visuospatial attention and working memory. The increased variability in RT which we found in children with FHR-SZ and FHR-BP is compatible with subtle impairments in these domains. For instance, a reduced ability to sustain attention across trials may increase the variability in RT. Notably, however, we would like to stress that we did not find any consistent group-wise differences in behavior and fMRI-based measures of executive control in our study. We have thus no reason to assume that a reduced ability to reorient spatial attention or reduced working memory capacity had a relevant detrimental effect on executive control during task-based fMRI. Therefore, we did not consider these cognitive domains in the statistical models of our fMRI analyses.

The cross-sectional design of the present study precludes any causal interpretations regarding risk of later development of BP and SZ. Longitudinal studies investigating the developmental trajectories of possible cognitive and/or neurobiological deficits are warranted to assess the state or trait nature of the (possible) early endophenotypic markers for SZ and BP. Our follow-up study, The VIA 15 study,^60^ will enable us to explore these aspects. Another important point is that children at FHR-SZ and FHR-BP in the present study were grouped based on the parent’s psychiatric history. Offspring of parents with either SZ or BP have not only increased risk of developing the specific disorder that is expressed by the parent, but also an increased risk for a broad range of other psychiatric disorders.^3,4^ Thus, we cannot conclude from the results of the current paper that the variability in the timing of responses distinguishing children at FHR-SZ and FHR-BP from PBC is specific to the risk for SZ vs BP itself or not. Further, the rate of conversion to SZ or BP in FHR cohorts is estimated at approximately 10%–40%.^61,62^ Hence, many of the included children will undergo development that does not result in SZ or BP. Therefore, the robustness of the effects must be relatively large to show statistical significance.

In conclusion, we found no differences in executive control or related brain activity in the largest cohort of 11- to 12-year-old children with FHR-SZ and FHR-BP compared to PBC. However, a main effect of group on the variability of timing of responses was observed indicating that, independent of the visuospatial response conflict, children with FHR-SZ and FHR-BP were more variable in the timing of their responses. This timing variability of responses during the task was also independent of past or present psychopathology, making this behavioral feature a possible endophenotypic trait marker for risk of SZ or BP. Longitudinal designs are warranted to illuminate developmental trajectories in individuals that may go on to develop SZ or BP.

## Supplementary Material

sbad134_suppl_Supplementary_Material

## Notes

Hartwig R. Siebner has received honoraria as speaker from Sanofi Genzyme, Denmark, Lundbeck AS, Denmark, and Novartis, Denmark, as consultant from Sanofi Genzyme, Denmark, Lophora, Denmark, and Lundbeck AS, Denmark, and as editor-in-chief (Neuroimage Clinical) and senior editor (NeuroImage) from Elsevier Publishers, Amsterdam, The Netherlands. He has received royalties as book editor from Springer Publishers, Stuttgart, Germany and from Gyldendal Publishers, Copenhagen, Denmark. During preparation of the manuscript, Ayna Baladi Nejad changed employment to Novo Nordisk A/S.
